# New insights into the genetic mechanism of IQ in autism spectrum disorders

**DOI:** 10.3389/fgene.2013.00195

**Published:** 2013-10-18

**Authors:** Harold Z. Wang, Hai-De Qin, Wei Guo, Jack Samuels, Yin Yao Shugart

**Affiliations:** ^1^Unit on Statistical Genomics, Intramural Research Program, National Institute of Mental Health, National Institutes of HealthBethesda, MD, USA; ^2^Department of Psychiatry and Behavioral Sciences, The Johns Hopkins University School of MedicineBaltimore, MD, USA

**Keywords:** GWAS, functional variants, rare variants, common variants, autism, cognitive development

## Abstract

Autism spectrum disorders (ASD) comprise a number of underlying sub-types with various symptoms and presumably different genetic causes. One important difference between these sub-phenotypes is IQ. Some forms of ASD such as Asperger’s have relatively intact intelligence while the majority does not. In this study, we explored the role of genetic factors that might account for this difference. Using a case–control study based on IQ status in 1657 ASD probands, we analyzed both common and rare variants provided by the Autism Genome Project (AGP) consortium via dbGaP (database of Genotypes and Phenotypes). We identified a set of genes, among them HLA-DRB1 and KIAA0319L, which are strongly associated with IQ within a population of ASD patients.

## INTRODUCTION

Autism gained recognition in the 1940s as a mental disorder characterized by social deficits, communication difficulties, and other abnormalities. Since then, scientists have increasingly recognized that autism is not one but a family of conditions that share certain clinical characteristics. Currently, classical autism, Asperger’s syndrome, Rett’s syndrome, childhood disintegrative disorder, and pervasive developmental disorder not otherwise specified (PDD-NOS) are grouped together as autism spectrum disorders (ASD). However, the recent revision of in the Diagnostic and Statistical Manual of Mental Disorders version 5 replaced this categorization with a continuous scale of severity (Halfon and Kuo, 2013).

There is considerable evidence for the role of inheritance in the etiology of autism and related disorders. Studies have consistently reported that the prevalence of autism in siblings of autistic children is approximately 15–30 times greater than the rate in the general population (Szatmari, 1999). More recently, identified genetic variants include inherited mutations, *de novo* mutations, single point mutations, and copy number variants (CNVs). In particular, researchers reported hundreds of ASD risk factors, ranging from *de novo* to inherited, CNVs to single point mutations (Anney et al., 2012).

Some variants found to be associated with ASD were discovered only when researchers restricted the study subjects to a specific population group. The distinction by IQ may be particularly relevant in ASD research, helping to separate Asperger’s syndrome, an ASD sub-type which spares language development, from autism, which does not. For example, in a recent study, Anney et al. (2012) identified a variant, rs1718101, which was strongly associated with ASD only in Europeans with high-IQ. In the current study, we hypothesized that the genetic etiology of ASD may be different based on IQ status. To test this hypothesis, we compared genotypic frequencies in high-IQ ASD probands with those of the low-IQ probands. We analyzed both common and rare variant. Specifically, we used the sequence kernel association test (SKAT) developed by Wu et al. (2011) to analyze the rare variants with minor allele frequency (MAF) less than 0.05.

## MATERIALS AND METHODS

### DATA DESCRIPTION

The study was conducted using a genome-wide association study (GWAS) data set of ASD families evaluated by the Autism Genome Project (AGP) consortium [provided by dbGaP (database of Genotypes and Phenotypes); Anney et al., 2012]. The AGP consortium represented more than 50 centers in North America and Europe. The centers collected clinical information from 2705 ASD families for the combined stage 1 and 2 study. Autism Diagnostic Interview-Revised (ADI-R) (2) and Autism Diagnostic Observation Schedule (ADOS) (3) were used for research diagnostic evaluation. Individuals were classified into “strict” or “spectrum” (i.e., includes strict) disorders, based on ADI-R and ADOS classification. Individuals with known karyotypic abnormalities, fragile X mutations, or other genetic disorders were excluded. Genotyping was performed by using the Illumina Human 1M-single Infinium BeadChip array (Anney et al., 2012). This resulted in 2665 ASD families (7880 individuals). We checked for Mendelian errors using PedCheck, and found none (O’Connell and Weeks, 1998). We further checked for per-individual genotyping missing rate, and removed those with more than 50%, leaving 7769 individuals within 2604 pedigrees. Because our research aim was to investigate the role of genetic variants associated with IQ difference in IQ in ASD patients, we focused on the probands and excluded their parents from this study.

### ANALYTICAL METHODS

High-IQ probands in the AGP data set were defined by the AGP committee as those with IQ greater than 80, while low-IQ probands were defined as those with IQ of between 25 and 70. Using this definition, out of 2095 probands with non-missing IQ statues included in the data, 1034 were classified as high-IQ, 623 as low-IQ, and 438 as normal-IQ. Probands with missing IQ statuses were not included in the analyses. In this paper, we compared the 1034 high-IQ probands to the 623 low-IQ probands for a total of 1657 individuals. Of these 1657 individuals, 918 high-IQ individuals and 511 low-IQ individuals for a total of 1429 were Caucasian. This required us to account for population stratification in this study.

Our approach differed for common and rare variants. We used MAF of 0.05 as the threshold to differentiate between the two types of variants. For common variants, we used PLINK’s (v1.07) built in function to account for population stratification. We first calculated the pair wise identity by state (IBS) matrix, and then performed a multidimensional scaling (MDS) analysis using two dimensions. We then used the two-dimensional MDS statistics along with sex as covariates to perform a logistic regression for each individual common single nucleotide polymorphism (SNP).

The analysis of rare variants is more complicated since, given the low numbers of informative individuals, association results for single rare variants tend to be unreliable. For this study, we used the SKAT (Wu et al., 2011). As with many other methods designed for rare variant analysis, SKAT analyzes multiple variants together as a unit. This remedied the lack of power for single rare variants by combining the effects of multiple variants. However, unlike the burden tests such as collapsing methods, which aggregate variants into a single variable before performing statistical regression, SKAT combines individual variant-test statistics after analyzing each variant independently. This is advantageous compared to collapsing methods when large numbers of variants affect the phenotype to increase or decrease the risk, and also when a large fraction of variants is non-causal. We used a gene-based method in our approach to rare variants, in which rare variants outside of known genes were not included in our analysis and the rest analyzed collectively via SKAT on a gene-by-gene basis. Dealing with population stratification via MDS analysis was not satisfactory for rare variants; thus, we included only Caucasian probands in this analysis.

## RESULTS

### POPULATION STRATIFICATION

**FIGURE 1:**
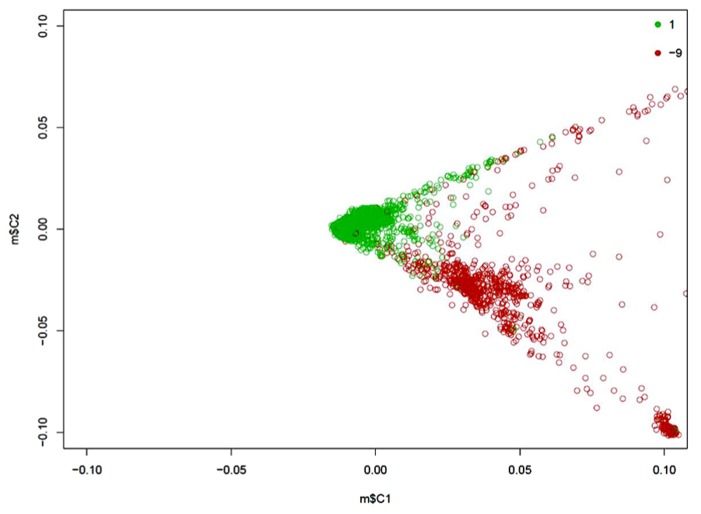
**Two-dimensional MDS plot of the AGP population.** The green circles are Caucasian individuals; the red circles are those of other ethnicities.

**FIGURE 2:**
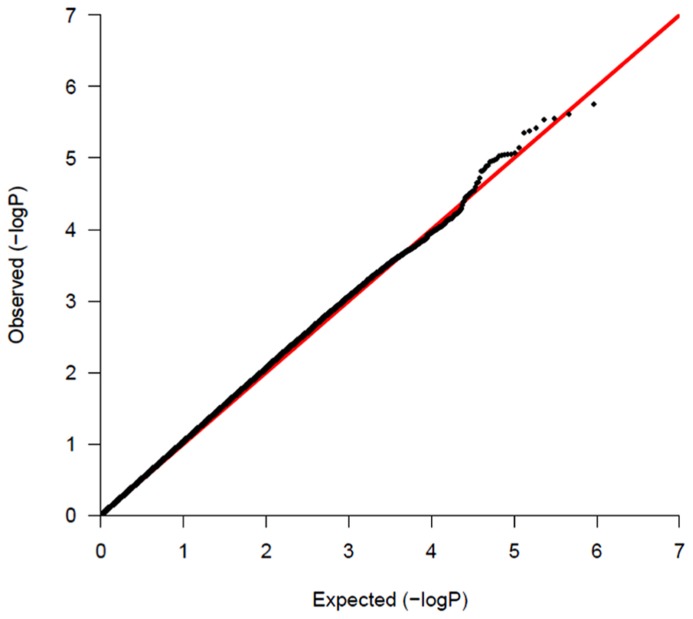
**QQ-plot of the *p*-values of common variant analysis**.

Of 1657 probands, 1429 are of Caucasian descent. The MDS plot obtained during the common variant analysis process is shown in **Figure 1**. Population stratification is significant for the sample. The Caucasian probands were relatively close genetically, while non-Caucasian individuals showed wide genetic differences among themselves. Specifically, non-Caucasians seemed to group themselves into two clusters. These could be different non-Caucasian ethnicities, but data were not available for proper identification. We presented a QQ-plot with the *p*-value of our adjusted analysis (**Figure 2**).

### COMMON VARIANTS

We analyzed a total of 878,930 SNPs. Fifteen SNPs had associations with *p*-value lower than 10^-5^, and 82 with *p*-values lower than 10^-4^ (data not shown). Forty-eight of the variants found in the high-IQ vs. low-IQ comparison have odds-ratio of less than 1, indicating an association with low-IQ, while the remainders are associated with high-IQ. We probed into the biological relevance of all SNPs with *p*-values lower than 10^-4^ in the NCBI SNP database, by analyzing genes that contain or are situated close to the SNP. Seventeen SNPs out of 192 in the high-IQ vs. low-IQ analysis fell within or near genes that have a significant role in the nervous system and neurodevelopment. The details are listed in **Table 1**.

**Table 1:** Common variant analysis results of high-IQ vs. low-IQ.

CHR	SNP	BP	Risk allele	TEST	Sample size	OR	STAT	*p*-Value	Gene
6	rs9268880	32539336	A	ADD	1657	0.7089	-4.443	8.85 × 10^-6^	HLA-DRB1
6	rs6903608	32536263	G	ADD	1656	0.7121	-4.391	1.13 × 10^-5^	HLA-DRB1
6	rs6923504	32536164	C	ADD	1656	0.7135	-4.365	1.27 × 10^-5^	HLA-DRB1
4	rs17012830	88670120	A	ADD	1650	0.6437	-4.242	2.22 × 10^-5^	SPARCL1
6	rs4715377	13622276	G	ADD	1656	1.809	4.176	2.97 × 10^-5^	GFOD1
2	rs10190416	36405331	G	ADD	1652	1.41	4.141	3.46 × 10^-5^	CRIM1
18	rs238129	3478151	A	ADD	1657	1.359	4.131	3.62 × 10^-5^	DLGAP1
17	rs12453363	45576919	A	ADD	1655	0.6792	-4.076	4.58 × 10^-5^	PPP1R9B
13	rs12872448	98393248	A	ADD	1653	0.6773	-4.041	5.31 × 10^-5^	DOCK9
7	rs805803	122842791	A	ADD	1653	1.449	4.032	5.52 × 10^-5^	IQUB
2	rs968796	3872434	G	ADD	1657	1.35	3.976	7.02 × 10^-5^	DCDC2C
10	rs10884381	108676055	G	ADD	1655	0.7411	-3.95	7.80 × 10^-5^	SORCS1
3	rs9289026	116838866	G	ADD	644	0.5493	-3.917	8.98 × 10^-5^	GAP43
8	rs1469039	140720961	A	ADD	1653	1.527	3.913	9.10 × 10^-5^	KCNK9
10	rs10786981	108671720	A	ADD	1657	0.7437	-3.907	9.33 × 10^-5^	SORCS1
17	rs8066520	24400717	A	ADD	1657	1.498	3.898	9.72 × 10^-5^	DCC

### RARE VARIANTS

We used the hg19 database as the standard for gene annotation. Excluding genes that do not have rare variants, we analyzed 8060 genes for high-IQ vs. low-IQ comparisons. The top 15 ranked genes are presented in **Table 2**. Genes that are functionally relevant to the nervous system and neurodevelopment are discussed below.

**Table 2:** Rare variant results of high-IQ vs. low-IQ.

Gene	*p*-Value	*N*. marker test
LTA4H	0.000132	1
STEAP2	0.000201	2
ALK	0.000268	29
ZMYM4	0.000303	5
LINC00550	0.000316	1
FKTN	0.000402	2
KIAA0319L	0.000536	4
TFAP2E	0.000639	1
NRD1	0.000659	7
SEMA6A	0.000662	9
ACAD11	0.000769	1
UBA5	0.000769	1
SLC16A4	0.000782	2
RAB3B	0.000991	1

**N*. marker test is the number of markers to test for an association after excluding non-polymorphic or high missing rates markers.*

## DISCUSSION

The AGP dataset consists of ASD probands and their parents sequenced using a GWAS platform. Its purpose is to explore the role of common variants in ASD by using a transmission disequilibrium test (TDT) approach. In this study, we focused on the probands themselves and excluded their parents. We speculated that by using a case-comparison design, we could potentially identify the specific variants that differentiate high- vs. low-functioning ASD individuals.

A total of 15 SNPs met the *p*-value threshold of 10^-5^ while 82 genes met the less stringent significance threshold of 10^-4^. We then examined the properties of genes that contain or are close to these SNPs using the NCBI database. We were particularly interested in genes known to be related to neurological disorders and neurodevelopment. These genes, as well as their related biological functions are summarized in **Table 3**.

**Table 3:** Summary of known biologically relevant genes found in common variant analysis.

Gene	Effect
HLA-DRB1	ASD
SPARCL1	Astroglial cells
gfod1	ADHD
crim1	CNS development
dlgap1	Schizophrenia
ppp1r9b	Dendritic spines
DOCK9	Bipolar
IQUB	Intelligence
dcdc2c	Neurogenesis
sorcs1	Memory
GAP43	Neurogenesis
DCC	Axon guidance

The most interesting finding is that three of the SNPs are included within the human leukocyte antigen (HLA) region on chromosome 6, very close to the gene HLA-DRB1, which was implicated in a paper by Torres et al. (2012) to be protective against ASD. All three of the SNPs (rs9268880, *p* = 8.85 × 10^-6^; rs6903608, *p* = 1.13 × 10^-5^; rs6923504, *p* = 1.27 × 10^-5^) near HLA-DRB1 are associated with lower IQ.

Among the remaining genes, there are three general categories. The first category includes genes related to neurodevelopment. One of these is the gene DCDC2C, a member of the doublecortin gene family, which has been implicated in neuronal migration, neurogenesis, and retina development through regulation of cytoskeletal structure and microtubule-based transport. Mutations in genes of this family have been implicated in epilepsy and developmental dyslexia, among other disorders (Dijkmans et al., 2010). Another gene of this class is GAP43, named growth associated protein 43 because it is expressed at high levels in neuronal growth cones during development and axonal regeneration, and considered a crucial component of regenerative response in the nervous system (Skene et al., 1986; Aigner et al., 1995). The third of these genes is DCC, which encodes a netrin 1 receptor that acts as a cue for axon growth and guidance (Forcet et al., 2002). The fourth gene, SPARCL1, has been implicated in multiple cellular processes during brain development. Specifically, SPARCL1 is prominently expressed in radial glia, where it terminate radial glial guided neuronal migration, and is further expressed in the proliferative ventricular zone (VZ) of the embryonic cortex (Weimer et al., 2008). Another gene, CRIM1 has also been implicated in central nervous system (CNS) development, possibly via growth factor binding (Kolle et al., 2000).

The second category contains genes that are related to neural function. PPP1R9B belongs to this category. This gene encodes spinophilin, which is a regulatory subunit of protein phosphatase-1 catalytic subunit (PP1) and is highly enriched in dendritic spines. Allen et al. (1997) suggested that spinophilin may serve as a neuronal targeting subunit for PP1 and might be responsive to neuronal inputs.

The third category contains genes linked to neurological conditions via bioinformatic methods, but has not yet been verified via biological experiments. These include GFOD1, which is associated with attention deficit hyperactivity disorder (ADHD), DLGAP1 which is associated with schizophrenia, DOCK9 associated with bipolar disorder, and SORCS1 which is associated with memory (Detera-Wadleigh et al., 2007; Lasky-Su et al., 2008; Reitz et al., 2011; Li et al., 2013). Interestingly, the SNP rs805803 is in close proximity (75 kb) to rs7791660, which was shown to be associated with mathematical ability (Docherty et al., 2010).

Considering rare variants, three genes are noteworthy. The first is ALK, which is an oncogene whose mutation also disrupts CNS development (de Pontual et al., 2011). The second is KIAA0319L located on chromosome 1, which has been identified as a candidate for dyslexia. This gene is expressed in the brain and, based on its structural similarities to the gene KIAA0319, has been suggested to play a role in neuronal migration (Couto et al., 2008). The third gene SEMA6A is expressed in developing neural tissue and is required for proper development of the thalamocortical projection (Leighton et al., 2001).

## CONCLUSION

In this study, we used a case–control approach to investigate the association of genetic variants with IQ in the ASD population. We analyzed common variants and rare variants separately and in different ways, using a standard case–control association test implemented in PLINK for common variants, and the SKAT for rare variants. Considering their previously reported biological roles, we were able to identify several genes that are plausible candidates for involvement in brain development in ASD patients. To our knowledge, this is among the first studies that addresses this issue.

These genes are biologically relevant to CNS and neurodevelopment based on published literature, the most prominent examples being the genes KIAA0319L and HLA-DRB1. These genes warrant further investigation of their properties, both in regard to their connection with intelligence and relationship to ASD.

We acknowledge that the findings reported are preliminary, and it is possible that at least some of the associated genes are false positives. Thus, further molecular validations are warranted.

## Conflict of Interest Statement

The authors declare that the research was conducted in the absence of any commercial or financial relationships that could be construed as a potential conflict of interest.

## References

[B1] Aigner L. Arber S. Kapfhammer J. P. Laux T. Schneider C. Botteri F. (1995). Overexpression of the neural growth-associated protein GAP-43 induces nerve sprouting in the adult nervous system of transgenic mice. *Cell* 83 269–278 10.1016/0092-8674(95)90168-X 7585944

[B2] Allen P. B. Ouimet C. C. Greengard P. (1997). Spinophilin, a novel protein phosphatase 1 binding protein localized to dendritic spines. *Proc. Natl. Acad. Sci. U.S.A.* 94 9956–9961 10.1073/pnas.94.18.9956 9275233 PMC23308

[B3] Anney R. Klei L. Pinto D. Almeida J. Bacchelli E. Baird G. Bolshakova N. (2012). Individual common variants exert weak effects on the risk for autism spectrum disorders. *Hum. Mol. Genet.* 21 4781–4792 10.1093/hmg/dds301 22843504 PMC3471395

[B4] Couto J. M. Gomez L. Wigg K. Cate-Crter T. Archibald J Anderson B. (2008). The KIAA0319-like (KIAA0319L) gene on chromosome 1p34 as a candidate for reading disabilities. *J. Neurogenet.* 22 295–313 10.1080/01677060802354328 19085271 PMC5381963

[B5] de Pontual L. Kettaneh D. Gordon C. T. Oufadem M. Boddaert N. Lees M. (2011). Germline gain-of-function mutations of ALK disrupt central nervous system development. *Hum. Mutat.* 32 272–276 10.1002/humu.21442 21972109

[B6] Detera-Wadleigh S. D. Liu C. Y. Maheshwari M. Cardona I. Corona W. Akula N. (2007). Sequence variation in DOCK9 and heterogeneity in bipolar disorder. *Psychiatr. Genet.* 17 274–286 10.1097/YPG.0b013e328133f352 17728666

[B7] Dijkmans T. F. van Hooijdonk L. W. Fitzsimons C. P. Vreuqdenhil E. (2010). The doublecortin gene family and disorders of neuronal structure. *Cent. Nerv. Syst. Agents Med. Chem.* 10 32–46 10.2174/187152410790780118 20236041

[B8] Docherty S. J. Davis O. S. Kovas Y. Meaburn E. L. Dale P. S. Petrill S. A. (2010). A genome-wide association study identifies multiple loci associated with mathematics ability and disability. *Genes Brain Behav.* 9 234–247 10.1111/j.1601-183X.2009.00553.x 20039944 PMC2855870

[B9] Forcet C. Stein E. Pays L. Corset V. Llambi F. Tessier-Lavigne M. (2002). Netrin-1-mediated axon outgrowth requires deleted in colorectal cancer-dependent MAPK activation. *Nature* 417 443–447 10.1038/nature748 11986622

[B10] Halfon N. Kuo A. A. (2013). What DSM-5 could mean to children with autism and their families. *JAMA Pediatr.* 167 608–613 10.1001/jamapediatrics.2013.2188 23645093

[B11] Kolle G. Georgas K. Holmes G. P. Little M. H. Yamada T. (2000). CRIM1, a novel gene encoding a cysteine-rich repeat protein, is developmentally regulated and implicated in vertebrate CNS development and organogenesis. *Mech. Dev.* 90 181–193 10.1016/S0925-4773(99)00248-8 10642437

[B12] Lasky-Su J. Neale B. M. Franke B. Anney R. J. Zhou K. Maller J. B. (2008). Genome-wide association scan of quantitative traits for attention deficit hyperactivity disorder identifies novel associations and confirms candidate gene associations. *Am. J. Med. Genet. B Neuropsychiatr. Genet.* 147B 1345–1354 10.1002/ajmg.b.30867 18821565

[B13] Leighton P. A. Mitchell K. J. Goodrich L. V. Lu X. Pinson K. Shcerz P. (2001). Defining brain wiring patterns and mechanisms through gene trapping in mice. *Nature* 410 174–179 10.1038/35065539 11242070

[B14] Li J. M. Lu C. L. Cheng M. C. Luu S. U. Hsu S. H. Chen C. H. (2013). Genetic analysis of the DLGAP1 gene as a candidate gene for schizophrenia. *Psychiatry Res.* 205 13–17 10.1016/j.psychres.2012.08.014 22940546

[B15] O’Connell J. R. Weeks D. E. (1998). PedCheck: a program for identification of genotype incompatibilities in linkage analysis. *Am. J. Hum. Genet.* 63 259–266 10.1086/301904 9634505 PMC1377228

[B16] Reitz C. Lee J. H. Rogers R. S. Mayeurx R. (2011). Impact of genetic variation in SORCS1 on memory retention. *PLoS ONE* 6:e24588 10.1371/journal.pone.0024588 PMC3202519 22046233

[B17] Skene J. H. Jacobson R. D. Snipes G. J. McGuire C. B. Norden J. J. Freeman J. A. (1986). A protein induced during nerve growth (GAP-43) is a major component of growth-cone membranes. *Science* 233 783–786 10.1126/science.3738509 3738509

[B18] Szatmari P. (1999). Heterogeneity and the genetics of autism. *J. Psychiatry Neurosci.* 24 159–165 10212560 PMC1188998

[B19] Torres A. R. Westover J. B. Rosenspire A. J. (2012). HLA immune function genes in autism. *Autism Res. Treat.* 2012 959073 10.1155/2012/959073 PMC3420779 22928105

[B20] Weimer J. M. Stanco A. Cheng J. G. Vargo A. C. Voora S. Anton E. S. (2008). A BAC transgenic mouse model to analyze the function of astroglial SPARCL1 (SC1) in the central nervous system. *Glia* 56 935–941 10.1002/glia.20666 18381651 PMC3642769

[B21] Wu M. C. Lee S. Cai T. Li Y. Boehnke M. Lin X. (2011). Rare-variant association testing for sequencing data with the sequence kernel association test. *Am. J. Hum. Genet.* 89 82–93 10.1016/j.ajhg.2011.05.029 21737059 PMC3135811

